# Role of NMDA Receptor-Mediated Glutamatergic Signaling in Chronic and Acute Neuropathologies

**DOI:** 10.1155/2016/2701526

**Published:** 2016-08-18

**Authors:** Francisco J. Carvajal, Hayley A. Mattison, Waldo Cerpa

**Affiliations:** ^1^Laboratorio de Función y Patología Neuronal, Departamento de Biología Celular y Molecular, Facultad de Ciencias Biológicas, Pontificia Universidad Católica de Chile, 8331150 Santiago, Chile; ^2^Department of Molecular Biology, Massachusetts General Hospital, Boston, MA 02114, USA

## Abstract

N-Methyl-D-aspartate receptors (NMDARs) have two opposing roles in the brain. On the one hand, NMDARs control critical events in the formation and development of synaptic organization and synaptic plasticity. On the other hand, the overactivation of NMDARs can promote neuronal death in neuropathological conditions. Ca^2+^ influx acts as a primary modulator after NMDAR channel activation. An imbalance in Ca^2+^ homeostasis is associated with several neurological diseases including schizophrenia, Alzheimer's disease, Parkinson's disease, Huntington's disease, and amyotrophic lateral sclerosis. These chronic conditions have a lengthy progression depending on internal and external factors. External factors such as acute episodes of brain damage are associated with an earlier onset of several of these chronic mental conditions. Here, we will review some of the current evidence of how traumatic brain injury can hasten the onset of several neurological conditions, focusing on the role of NMDAR distribution and the functional consequences in calcium homeostasis associated with synaptic dysfunction and neuronal death present in this group of chronic diseases.

## 1. Introduction

*Relevance of NMDA Receptor in Calcium Homeostasis: Structure and Properties*. In the central nervous system of mammals, *α*-amino-3-hydroxy-5-methyl-4-isoxazolepropionic acid (AMPARs) and N-methyl-D-aspartate ionotropic glutamatergic receptors (NMDARs) primarily mediate fast excitatory neurotransmission. Characteristic properties, including high Ca^2+^ permeability, allow NMDARs to play a critical role in brain development, neuropathology, and synaptic plasticity [1]. In addition, NMDARs seem to play a structural role at the synapse by recruiting scaffolding and signaling complexes through their intracellular domains [2, 3]. The number, properties, and subunit composition of synaptic NMDARs are critical for proper synaptic functioning and maintaining the integrity of the synapse, regulating calcium influx and different signaling cascades associated with receptor activation [4]. On the other hand, deregulation in the appropriate influx of Ca^2+^ through NMDARs contributes to neuronal death in acute brain injury, including stroke and ischemia, but also contributes to neuronal loss underlying several neurodegenerative diseases such as Alzheimer's disease (AD) and Huntington's disease (HD) [5].

Structurally, the NMDARs are tetraheteromeric channel pores formed by the obligatory GluN1 subunit, plus GluN2 or GluN3 subunits. These subunits contain several variants: GluN1 subunits with eight splice variants, four GluN2 subunits (GluN2A–D), and two GluN3 subunits. Each subunit has, structurally, an extracellular N-terminal, a reentrant loop that forms the channel pore, and an intracellular C-terminal. Functional NMDARs, in the forebrain of the CNS, are formed by two GluN1 subunits and two GluN2/3 subunits [6]. Glutamate binds the GluN2 subunit while D-serine and glycine, two coagonists, bind the GluN1 subunit in synaptic and extrasynaptic sites, respectively [7]. The domain for Mg^2+^ block and Ca^2+^ permeability is in the pore formed by the internal loop [6]. GluN2 subunits give specific and key biophysical and pharmacological properties, including sensitivity to glutamate, protons, polyamines, and Zn^2+^, modulation by glycine, Ca^2+^ permeability, and differential channel kinetics, including deactivation time and open probability [8]. GluN2 subunits control the trafficking and delivery of NMDARs to the plasma membrane and synaptic compartments through their intracellular domains [6].

In brain structures such as the brain stem, hippocampus, and neocortex, the ratio of GluN2A/2B increases during early postnatal development [9]. For example, in the hippocampus, the selective GluN2B inhibitor, ifenprodil, shows an age-dependent decrease in the effect of NMDAR blockade in rats between postnatal day 1 (P1) and young adult (~P21) [10]. Changes in the GluN2A/2B ratio can be estimated by measuring mRNA [11, 12] or protein levels [13]. The predominant GluN2 subunits in the mammalian forebrain, 2A and 2B, also control the trafficking of NMDARs with subunit specific rules: GluN2A subunits appear to be more abundant in synaptic sites while GluN2B is present in both synaptic and extrasynaptic domains. GluN2B-containing receptors have faster rates of diffusion than GluN2A-containing receptors, which contributes to the enrichment of GluN2A at mature synaptic sites [14]. Thus, GluN2B-containing receptors are inserted into the synapse in an activity-independent or constitutive manner. In contrast, incorporation of GluN2A-containing receptors requires synaptic activity and they accumulate intracellularly when activity is blocked [15].

The toxic effects of glutamate were described for the first time several years ago with the discovery that the application of L-glutamate caused toxicity in the inner layers of the retina. In this study, glutamate was injected in neonatal and adult mice daily. In P4 mice, glutamate injection led to the reduction to half of the inner nuclear layer and a significant reduction of the ganglion-cell layer after 2–4 days of treatment compared with control animals. In adult mice, the effect of glutamate injection had similar effects to those in P4 mice within minutes of injection, including a dramatic reduction of the inner nuclear layer of retina and the ganglion-cell layer [16]. In 1969, the term excitotoxicity was coined [17]. Using subcutaneous injection of glutamate in postnatal mice, the authors demonstrated that acute neuronal necrosis in several regions of the brain was induced by excitotoxicity [17]. In an anoxia model, using hippocampal neurons* in vitro*, cells treated with a postsynaptic blocker of excitatory amino acid (g-D-glutamylglycine) exhibited decreased neuronal death compared to untreated neurons. This protective effect also works in neurons treated exogenously with glutamate [18]. Similarly, the selective antagonist of NMDARs substantially attenuated neuronal injury in cultured cortical neurons [19], including 2-amino-7-phosphonoheptanoate (APH), 2-amino-5-phosphonovaleric acid (APV), and phencyclidine (PCP). In addition to the acute effects of glutamate, several chronic diseases appear to have an excitotoxic component. These diseases include Alzheimer's disease (AD), Parkinson's disease (PD), amyotrophic lateral sclerosis (ALS), and Huntington's disease (HD).

The plasma membrane sodium-calcium exchanger (NCX) is one of the most important proteins that functions to maintain the physiological concentration of calcium in the cell [20]. Working with the driving force from Na^+^ influx, this transporter extrudes Ca^2+^ in normal conditions. However, the transporter is affected by the overactivation of NMDARs and subsequent Ca^2+^ overload after excitotoxic stimulation [21]. Excitotoxicity results in a significant increase in Ca^2+^ influx primarily from open NMDAR channels that cause a secondary rise in the intracellular Ca^2+^ concentration. This secondary overload is correlated with neuronal death induced by calcium overload [22]. An imbalance in Ca^2+^ homeostasis is produced in several neurological conditions including epilepsy, AD, PD, HD, ALS, stroke, and traumatic brain injury (TBI). It is a common mechanism of toxicity, which could be a direct consequence of alterations in NMDARs distribution generating synaptic dysfunction and neuronal death.

*Synaptic versus Extrasynaptic Distribution and Associated Signaling*. NMDARs are more dynamic than originally assumed, with neurons able to regulate the amount, distribution, and subunits composition of synaptic and extrasynaptic NMDARs [7, 8, 15]. However, the signals and mechanisms controlling the presence of NMDARs in different domains are dependent on the phosphorylation state of the GluN2B subunit [23]. Phosphorylation of tyrosine 1472 of GluN2B is critical to maintain NMDARs at the synapse and prevent endocytosis, whereas phosphorylation of GluN2B at tyrosine 1336 is associated with enrichment of extrasynaptic NMDARs [23]. Both phosphorylation sites are substrates of a Src-family kinase, Fyn [24]. Moreover, the phosphorylation state of NMDARs is modulated by striatal-enriched protein tyrosine phosphatase (STEP) by two parallel pathways: direct dephosphorylation of GluN2B-Tyr 1472 [23, 25] and indirectly via dephosphorylation and inactivation of Fyn [26]. On the other hand, STEP is differentially regulated by synaptic and extrasynaptic NMDARs [24]. These receptors are localized in distinct compartments of the neuronal membrane where they initiate signaling pathways when activated by glutamate [27, 28]. The signaling pathways downstream of NMDAR stimulation involve multiple proteins (Table 1), only some of which will be highlighted here. Synaptic activation of NMDARs activates a signaling cascade that includes ERK activation via MEK1 [29, 30], but activation of synaptic and extrasynaptic NMDARs promotes activation followed by inactivation of ERK signaling [31, 32]. The mechanism includes activation through ERK phosphorylation by synaptic stimulation of NMDARs, or ERK dephosphorylation and inactivation by stimulation of extrasynaptic NMDARs [28]. Activation of ERK through synaptic NMDARs [27, 29] induces phosphorylation and activation of the transcription factor CREB [33]. The calcium influx through synaptic NMDARs involves MEK1 activation and finally ERK1/2 activation [32]. In contrast, extrasynaptic NMDARs stimulation inhibits the CREB pathway through dephosphorylation of its activation site [34]. Additionally, only synaptic NMDAR stimulation is associated with the activation of the PI3K/akt kinase signaling pathway [35].

On the other hand, inactivation of ERK (by extrasynaptic NMDA receptor stimulation) is associated with calpain activation and STEP cleavage [36]. Ca^2+^ influx through extrasynaptic NMDARs involves binding of the phosphatase STEP to the channel, with direct inhibition of ERK 1/2. Another key player, CaMKII, appears to have a role in the activation of both pathways (synaptic and extrasynaptic NMDARs activation) in part because of its multifunctional properties [37]. An interesting target for ERK 1/2 is the protein known as Jacob [38]. Jacob works by translocating to the nucleus in a phosphorylated or nonphosphorylated form. However, only the phosphorylated form is associated with an elevated level of phosphorylated form of CREB (p-CREB), Arc 3.1, and BDNF while the nonphosphorylated form of Jacob is associated with low levels of a transcriptionally active form of CREB, p-CREB [38, 39]. Jacob is phosphorylated by ERK1 at Ser 180 after induction of synaptic activity, including LTP (but not LTD). Translocation of the nonphosphorylated form of Jacob to the nucleus is associated with the deleterious events in the synapse that subsequently cause cell death in rat brain [38]. In the last few years a unified hypothesis placed in tandem “subunit hypothesis” (GluN2B subunits associated toxicity) with the “localization hypothesis” (extrasynaptic NMDARs associated with toxicity) to link controversial results [40]. A good example for this point is experiments in neuronal culture and transgenic mice where C-terminal of GluN2 subunits (A and B) were swapped to determine the participation of this domain in NMDA-induced toxicity, with C-terminal of GluN2B (other domains of GluN2A) exhibiting stronger physical/functional coupling to the PSD-95-nNOS pathway, suppressing protective CREB activation [41].

## 2. Role of NMDARs in Traumatic Brain Injury and Other Acute Damage

### 2.1. Traumatic Brain Injury, Relevance, and Mechanism: Glutamatergic Role

TBI is the result of mechanical external force including contusion, fast acceleration, and expansive waves that produces temporal or permanent cognitive damage and triggers physical and psychosocial alterations including headache, memory problems, attention deficits, difficulty thinking, mood swings, and frustration [51, 52]. On the global level, TBI is a critical health problem, constituting a major cause of death and disability among young adults [52], with a high cost to society due to long years of disability or death. In the USA, it is estimated that around 1.7 million of cases of TBI occur annually [53]. In Europe, TBI is among the top three causes of injury-related medical costs [54]. The number of cases of TBI is rising sharply and the main reason is due to increasing motor-vehicle use in less developed countries [55]. In more developed countries, the majority of cases of TBI are caused by falls in older adults [56]. TBI is a complex conditions where structural and functional damage is a result of both primary and secondary injury [57]. The primary injury occurs as a result of external force and can result in hemorrhage, tissue, and axonal damage. The secondary lesion progresses from minutes to months after the primary lesion, generating activation of metabolic cascades including cellular and molecular changes such as excitotoxicity, inflammation, oxidative damage, and synaptic injury [58, 59].

An important cause of damage leading to the secondary injury is neuroinflammation due to the loss of astrocytes, which regulate the availability of glutamate at the synapse. Glutamate transporters GLT-1 and GLAST present in glial cells regulate the extracellular glutamate and limit excitotoxicity by clearing off excess glutamate [60]. Part of the loss of functionality of astrocytes is triggered by inflammatory cytokines including TNF-*α*, which is found at high levels in cerebrospinal fluid within 24 h of brain trauma [61]. Astrocytes control the excess of glutamate from spillover, and when their functionality is compromised, the effect of excitotoxicity is exacerbated after TBI. Astrocytes also influence and regulate neuronal excitability [62], neurotransmission [63, 64], and plasticity in glutamatergic synapses [60], but the mechanisms underlying their role in TBI have not been explored.

The central mechanism underlying TBI is glutamate excitotoxicity (or toxicity induced by glutamate) and intracellular calcium overload that triggers biochemical cascades that lead to synaptic damage and neuronal death [65–67]. TBI induces an increase in the expression of GluN1, GluN2A, and GluN2B subunits of NMDARs in the hippocampus of mice [68]. However, whether these NMDARs are synaptic or extrasynaptic was not explored. There is no functional or* in vivo* evidence of the role of extrasynaptic NMDARs in TBI. However, a recent study found that extrasynaptic NMDARs are overactivated as a result of stretch injury in cortical cell culture [69], suggesting that extrasynaptic NMDAR activation may be potential therapeutic targets for preventing secondary lesions in TBI.

In addition to excitotoxic and inflammatory consequences of TBI, the oxidative stress associated with secondary damage has important consequences on the functionality of neurons [65] with an important role of mitochondrial dysfunction [70]. Oxidative stress could have consequences not only for neuronal survival but also because synaptic plasticity is affected under oxidative conditions [71, 72], becoming another interesting target of intervention in TBI.

Neuronal loss and synaptic alterations are common characteristics in a wide spectrum of neurological diseases, including PD and AD where the most striking symptom is memory loss. Thus, it is not surprising that the brain areas that are essential for learning and memory, such as the hippocampus and neocortex [73], are affected in these pathological conditions. TBI shares with these diseases the decrease in neuronal population and the number of synapses: cortical contusion results in a decline in the number of total synapses and the total neurons in the hippocampus of rats subjected to TBI [74]. In mice, moderate TBI is able to decrease the measure of dendritic branching three days after impact [67]. TBI also induces a deficit in spatial learning that lasts for the 90 days of the experiment as measured by escape latency (using water maze) at 7, 30, 60, and 90 days after TBI [75].

The levels of excitatory amino acids, especially glutamate, increase in the extracellular space after TBI [76] activating the toxic mechanisms of excitotoxicity. Functional consequences in TBI models include an impairment in synaptic plasticity measured by the inability to induce long-term potentiation (LTP) in rat hippocampal slices [77, 78] and cognitive impairment in spontaneous exploration and spatial memory [79–83]. Alterations in NMDARs caused by neuronal injury make the system more vulnerable to damage induced by glutamate [84–86] by a mechanism dependent on Ca^2+^ concentration. The dynamic of alterations of NMDARs has two stages: the level of receptors is transiently diminished in the hippocampus after TBI [87], but cultured neurons show an increase in extrasynaptic GluN2B-containing NMDARs after mechanical stretch [69]. Thus, it is possible that TBI could alter the neuronal Ca^2+^ dynamics primarily through extrasynaptic NMDAR stimulation.

The ionotropic glutamate receptor AMPA is also compromised in TBI conditions. After TBI, a reduction in AMPARs desensitization is observed [88, 89]. Other lines of evidence shows that TBI promotes GluR2 phosphorylation and internalization and enhances expression of Ca^2+^-permeable AMPARs in the hippocampus [88, 89]. The phosphatase and tensin homolog (PTEN) target for inhibition attenuates the death of hippocampal neurons after injury by decreasing the translocation of GluR2 subunits to the membrane, similar to effect of blocking GluR2-lacking AMPARs, both* in vitro* [90]. The same PTEN is activated by stimulation of extrasynaptic NMDARs [91]. It is possible to find direct cross-talk between the effects of TBI on both AMPARs and NMDARs expanding the glutamatergic target of TBI.

## 3. Ischemia/Reperfusion

**Figure 1:**
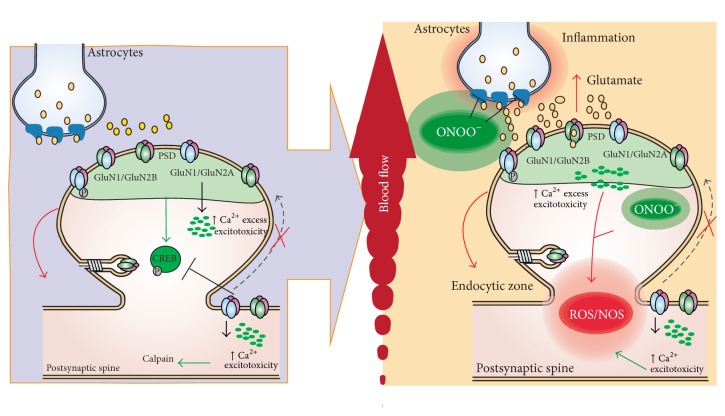
Implications of NMDAR in acute damage, ischemia/reperfusion. During ischemia, the overactivation of STEP induces the internalization of NMDARs, principally of GluN2B subunits, and the activation of extrasynaptic NMDAR triggers an excess of Ca^2+^ influx and excitotoxic events related to decreases in CREB activation and increases in calpain activity. During reperfusion, injury induces the generation of ROS and ONOO^−^. The increase of ONOO^−^ alters the activity of glutamate transporter in astrocytes. The excess glutamate leads to the overactivation of NMDARs.

Ischemia/reperfusion injury is the tissue damage caused when the blood supply returns to tissue following a period of ischemia. The reintroduction of molecular O_2_ into ischemic tissue upon reperfusion leads to the overproduction of reactive oxygen species (ROS). A cascade of ROS formation is initiated by the generation of O^2•−^, which is generated by NADPH oxidase (NOX). NO and O^2•−^ may react together to produce significant amounts of a much more oxidative active molecule, peroxynitrite (ONOO^−^), which is a potent oxidizing agent that causes posthypoxic cellular injury [92]. ONOO^−^, formed from the diffusion-controlled reaction of O^2•−^ with NO, is a highly toxic ROS. It has been proposed that a number of the toxic effects of NO are due to the subsequent generation of ONOO^−^ [93] (Figure 1). ONOO^−^ is cytotoxic via several mechanisms, including the initiation of lipid peroxidation, the direct inhibition of mitochondrial respiratory chain enzymes, the inactivation of membrane sodium channels, the modifications of oxidative proteins, and the inhibition of antioxidant enzymes [94]. Due to its toxic nature, ONOO^−^ may be involved in a number of inflammatory conditions [95, 96], cardiovascular diseases [97], and neurodegenerative diseases [98]. ONOO^−^ has been implicated in several pathophysiological pain processes, such as thermal hyperalgesia associated with inflammation and nerve injury [99], opioid-induced hyperalgesia and antinociceptive tolerance [100], and spinal activation of NMDARs [101]. Increased NMDAR activity in the spinal cord, as detected by increased phosphorylation of the GluN1 subunit, is critically involved in the development of central sensitization of chronic pain [102, 103]. Furthermore ONOO^−^ is thought to contribute to central sensitization through the alteration of NMDAR activation by nitrating proteins that are important in the maintenance of normal nociceptive processing, such as MnSOD [104], glutamate transporters, and glutamine synthase [105, 106]. The ONOO^−^-mediated nitration of SOD inactivates the enzyme, which results in increased O_2_ and ONOO^−^ levels, leading to enhanced postsynaptic neuronal responsiveness that contributes to central sensitization [101, 107]. Nitration of glutamate transporters and glutamine synthase disrupts glutamate homeostasis and increases glutamate neurotransmission, and the resulting signaling events underlie central sensitization [105]. When glutamate transporters are nitrated by ONOO^−^, their inactivation results in increased glutamate concentrations and altered synaptic transmission [105] (Figure 1). Glutamine synthase, which catalyzes the conversion of glutamate and ammonia to glutamine, is also inactivated via nitration by ONOO^−^ [106]. Thus, ONOO^−^ is critically involved in the pathogenesis of ischemia/reperfusion injury-induced neuropathy, which is formed in the spinal cord in response to NMDAR activation and contributes to the development of central sensitization.

## 4. Stroke

Stroke is a major cause of death and disability in developed countries. Because neuronal death in the brain following stroke is an active and prolonged process [108], understanding the underlying death-signaling mechanisms can lead to therapeutics that minimize stroke damage even when administered several hours to days after a stroke. There are probably many mechanisms that underlie stroke damage, with NMDARs-mediated excitotoxicity being a primary factor [109, 110]. Not only has excessive NMDARs activation been considered a common pathological event leading to neuronal death in many neurological disorders [111], it also has a central role in ischemic neuronal death following stroke. Indeed, NMDAR blockers protect neurons from ischemic neuronal injuries in both* in vitro* and* in vivo* models [109, 111, 112].

In the adult forebrain, where stroke most frequently occurs, GluN2A receptors and GluN2B receptors are preferentially localized at synaptic and extrasynaptic sites, respectively [6, 113–115]. The “NMDAR location” and “NMDAR subtype” hypotheses are highly correlated [88, 89]. Notably, stimulating synaptic and extrasynaptic NMDARs would predominantly activate GluN2A receptors-dependent neuronal survival and GluN2B-mediated neuronal death pathways, respectively. Normal synaptic transmission activates predominantly GluN2A receptors, resulting in the maintenance of neuronal survival via the activation of the neuronal survival-signaling complex (NSC) immediately downstream of these receptors such as the cyclic-AMP response element-binding protein (CREB) signaling pathway [116–118], phosphoinositide 3-kinase (PI3K) [119], and kinase-D-interacting substrate of 220 kDa (Kidins220) [120].

**Figure 2:**
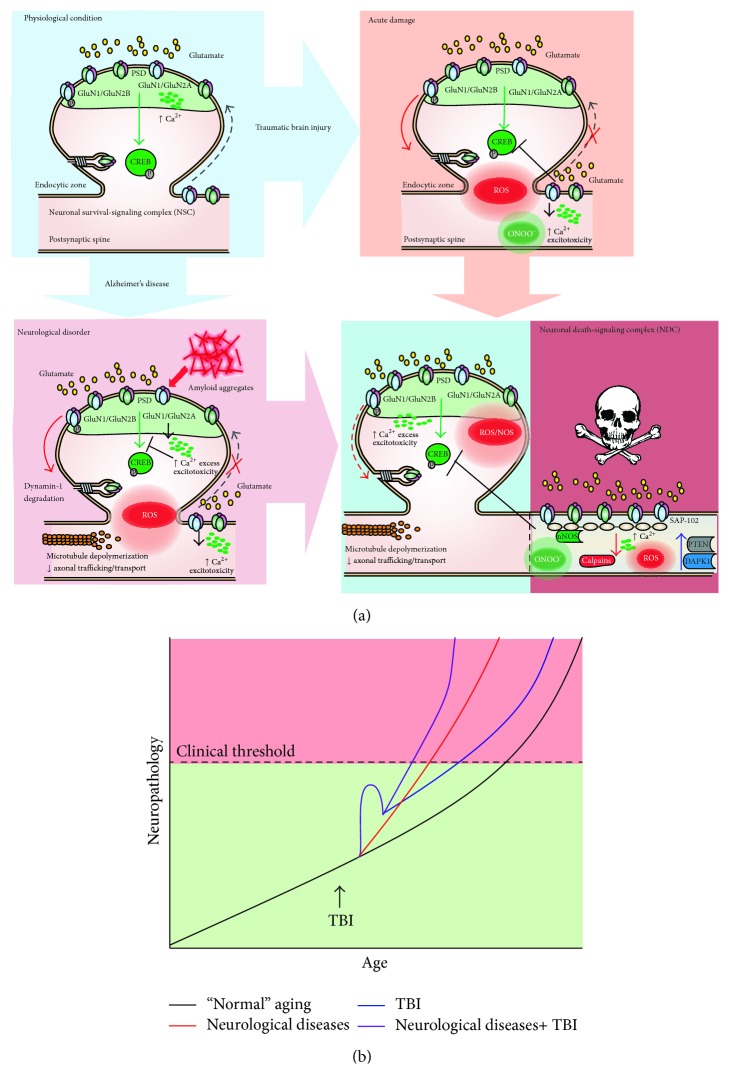
Dysregulation of NMDARs performance in neuropsychiatric disorders and in acute damage. (a) Schematic of the role of NMDARs in TBI and neurodegenerative disease. Under physiological conditions, synaptic NMDARs are activated as well as antiapoptotic cell pathways preventing excitotoxicity by targeting CREB. After acute damage, including TBI, there is a decrease in CREB activation, increased activation of extrasynaptic NMDARs, and ROS/NOS generation. In neurological disorders such as AD, there are alterations in cell signaling due to misfolding proteins, microtubule depolymerization, excessive Ca^2+^ influx, ROS generation, and excitotoxicity. The cell death mechanisms associated with glutamatergic transmission include calpains, PTEN, and DAPK1. (b) Hypothesized interaction between TBI, neurological diseases, and “normal” aging. The progression curves show the age of patients at disease onset and the severity of neurological symptoms. The black line shows the progression of neurodegeneration in normal aging and the red line shows the acceleration of neurodegeneration that occurs in diseases such as AD. This neurodegeneration includes neuroinflammation, oxidative stress markers accumulation, and the aggregation of misfolded proteins. This neurodegeneration can be accelerated after TBI both in “normal aging” (blue line) and in patients with neurodegenerative disease (purple line).

In hippocampal slices in which ischaemia was induced pharmacologically, glutamate surges were observed as a result of the reverse operation of the glutamate transporters [121]. Glutamate spillover to extrasynaptic sites preferentially stimulates GluN2B-containing receptors that mediate death by the activation of the neuronal death-signaling complex (NDC) associated with these receptors In this complex, PSD95 acts as a scaffolding protein to bring nNOS (neuronal nitric oxide synthase) into close proximity to the channel pore of GluN2B-containing receptors. This allows the efficient activation of nNOS by Ca^2+^ influx entering the channel pore, resulting in the NMDAR-mediated production of the highly neurotoxic molecule NO [122–126]. Other proteins that have been identified in the NDC include death-associated protein kinase 1 (DAPK1), a death-signaling protein [127] that is recruited to the NDC via its interaction with GluN2B following stroke challenge [128], PTEN [129], a well-characterized cell death-promoting molecule that was recently identified as a crucial component of the NDC [91, 130], and finally calpains, a family of Ca^2+^-activated cysteine proteases that plays a major role in translating the Ca^2+^ influx of NMDARs into neuronal damage [120, 131, 132] (components shown in Figure 2). Here, we loosely define the NSC and NDC to include all neuronal survival- and death-signaling proteins that closely associate with the NMDAR channel pore either through spatial compartmentalization at the synapse, in extrasynaptic sites, or through direct or indirect protein-protein interactions with NMDARs [113]. Considering that some of these signaling pathways are activated downstream of NMDAR activation, interventions that target these pathways may provide a longer therapeutic window for stroke treatment.

## 5. TBI as a Risk Factor for Neurological Disease: Role of NMDARs

It is possible to make a distinction between chronic traumatic encephalopathy (CTE) where a progressive neurodegenerative disease occurs in association with repeated trauma (examples include athletes and military personnel) and a single acute event of brain trauma (usually due to accidents). Here we will focus on how single traumatic events are able to accelerate the progression of chronic mental conditions. Several neurological conditions and how TBI contributes to these conditions will be described. Not all of the conditions are associated with TBI, but all of these pathologies share a common mechanism of toxicity mediated by glutamate. A wide range of evidence indicates that TBI is a risk factor associated with the onset and progression of AD and PD, but other neurodegenerative diseases such as HD and ALS have very few clinical reports of an aggravating event [88, 89, 133]. The secondary damage associated with TBI shares the molecular mechanism of damage associated with this group of neurological diseases. More general characteristics of TBI have been described earlier in this review.

### 5.1. Alzheimer's Disease

AD is the most common form of dementia and the most prevalent neurodegenerative disease in the elderly population [134]. AD progression has been associated with the selective loss of neurons in the hippocampus and neocortex, brain areas involved in memory and cognition. AD is characterized by synaptic loss, abnormal amyloid-beta peptide (A*β*) processing of A*β* precursor protein (APP), and hyperphosphorylation of tau, a microtubule associated protein. High levels of intracellular A*β* and the accumulation of the secreted form are believed to be a central causative factor for neurodegeneration in AD [135]. One of the neurotransmitter systems most affected in AD is the glutamatergic system and specially the transmission mediated by NMDARs [136]. Several reports indicate that the activation of NMDARs by A*β* accumulation may occur at early stages of the disease [137]. A recent study demonstrated that A*β* oligomeric species specifically activate GluN2B-containing NMDARs causing an immediate rise in calcium in cultured cortical neurons, producing unbalance of calcium homeostasis [138] (Figure 2(a)). Pharmacological inhibition of GluN2B-containing NMDARs by ifenprodil demonstrates that increases in Ca^2+^, induced by A*β* oligomers, are mainly mediated by this subunit [138]. One of the pharmacological treatments for AD approved by the Federal Drug Admin (FDA), memantine, is a noncompetitive open channel NMDAR blocker and has been primarily prescribed as a memory-preserving drug for moderate- to late-stage AD patients [139]. Memantine binds NMDARs with low affinity, which preferentially antagonizes NMDARs that have been excessively activated. Due to its relatively fast off-rate memantine does not substantially accumulate in the channel to interfere with synaptic transmission. Importantly, memantine has been shown to be well tolerated and safer than other nonselective NMDAR antagonists. Extrasynaptic NMDARs have been largely associated with NMDAR excitotoxicity in AD which may explain the therapeutic effects of memantine, which targets extrasynaptic NMDARs rather than synaptic NMDARs in the same neuron. This may also explain why memantine is well tolerated. Interestingly, it has been reported that Mg^2+^, an endogenous NMDARs blocker that binds near the memantine binding site at physiological concentrations, decreases memantine-mediated inhibition of GluN2A and GluN2B-containing receptors, while it has no effect on memantine-mediated inhibition of GluN2C and GluN2D-containing receptors. This suggests that the hypothesized mechanism of action for memantine should be reviewed in order to reconsider potential roles of GluN2C and GluN2D subunits [140]. However, taking into account that the NMDARs in the brain areas affected in AD are mainly composed of GluN2A and GluN2B subunits, this last observation may not be so relevant for the action of this compound in AD [136]. The use of memantine as an FDA-approved pharmacological therapy for AD demonstrates the success of treatments that regulate glutamatergic transmission and indicates that other antagonists that target NMDARs may be used to treat symptoms of AD; for example, ifendropil, a selective GluN2B subunit antagonist, could be used to prevent synaptic dysfunction in AD models [141, 142]. Supporting this idea, ifenprodil and MK-801 (a pore channel NMDAR inhibitor) were able to prevent downregulation of PSD-95 and synaptophysin levels induced by A*β*1–42 oligomers treatment [141], demonstrating that a selective pharmacological regulation of glutamatergic transmission is a good start in the search for a drug target to treat AD.

Recent studies have shown that long-term survivors of just a single moderate-to-severe TBI exhibited abundant and widely distributed neurofibrillary tangles (NFTs) and A*β* plaques in approximately one-third of the cases, but this was exceptionally rare in uninjured controls [143]. Surprisingly, the plaques found in TBI patients are strikingly similar to those observed in the early stages of AD [144]. Such findings demonstrate the long-term consequences of a single TBI event [145]. Another study where TBI patients underwent minimental state examination, apolipoprotein E genotyping, and amyloid-PET found an increase of amyloid accumulation and allele frequency of APOE4 in the mild TBI patients with cognitive impairment [146]. In transgenic mice models of AD, several studies have investigated A*β* after experimental brain injury in transgenic mice, reporting both increased and decreased plaque loads [147, 148]. Other additional main players in neurodegeneration observed in AD are the intracellular aggregates formed by the hyperphosphorylated form of tau [149]. Tau is critical for A*β* neurotoxicity [150]. A*β* is unable to induce toxicity in the absence of tau [150]. The relationship of A*β* toxicity mediated by tau through NMDARs was determined in organotypic hippocampal cultures from A*β* transgenic mice combined with viral expression of human wild type-tau protein (hTau) [151]. A*β* mice express human APP with the combined Swedish and Arctic mutation [152] and show intracellular A*β* deposit and behavioral deficit in Y-maze and water maze [152]. Overexpression of hTau in A*β* slices increases the synaptic damage observed in A*β* animals [151]. The deleterious synaptic effects in arcA*β* animals overexpressing hTau are prevented using the GluN2B-containing NMDAR antagonist ifenprodil. In contrast, the antagonist PEAQX (for GluN2A-containing NMDARs) does not prevent synaptotoxicity [151].

In terms of NMDAR distribution and whether synaptic or extrasynaptic stimulation is associated with AD, the phosphorylation of Jacob is inhibited and the nonphosphorylated form is translocated to the nucleus after A*β* treatment [39]. This effect is associated with a decrease in CREB phosphorylation and BDNF levels [38]. Using the antagonist ifenprodil in hippocampal cell cultures treated with A*β*, the amyloid toxicity is prevented, modulating Jacob-CREB signaling [142], indicating that GluN2B-containing NMDARs play a central role in the pathology of A*β* neurotoxicity.

In AD and other chronic mental conditions, the imbalance in Ca^2+^ homeostasis is controlled by the dynamics of NMDARs. Internal risk (genetic) factors, but also external modulators (diet, exposition to toxic environment, or accidents) could affect this tight regulation. Acute conditions, including traumatic brain injury, are able to dysregulate Ca^2+^ homeostasis and increase susceptibility to present these conditions (Figure 2(a)). A study with 649 participants found that self-reported head injury is associated with earlier onset of and increased risk for cognitive impairment and dementia, the presence of AD-type pathological changes, and increased risk of mortality [153]. Accumulation of A*β* is mediated by TBI which was shown in a study of 152 postmortem TBI brains in a wide range of ages (8 weeks–85 years old) and time after the TBI events (4 hours to 2.5 years) compared with a control group (51 to 80 years old). The presence of A*β* positive cases was higher for the TBI group with a 30% of the cases (46 of 152). Patients older than 60 years made up 50% of positive cases for A*β* in control conditions but this number increases to 70% in injured patients [154]. Tau pathology is also increased in TBI: in a study with 39 cases of TBI (death between 1 and 47 years after injury) compared with 47 control cases showed that 34% of patients under 60 affected by brain injury had tau pathology compared to only 9% of controls [143] (Figure 2).

### 5.2. Schizophrenia

Schizophrenia is a debilitating mental disorder affecting approximately 1% of the global population. The disorder has three clinical symptoms: episodic psychosis (hallucination, delusion), chronic withdrawal (negative symptoms), and pervasive cognitive deficits. There are also psychophysical abnormalities that may be the underpinnings of these clinical symptoms. Psychosis is treated with a broad class of antipsychotic medications that act by inhibiting the dopamine receptor, but this treatment causes severe motor and behavioral side effects and does not prevent the cognitive deficits [155].

Deficits in cognitive performance and behavioral manifestations (social withdrawal, increased motor stereotypy, and locomotor activity) of schizophrenia in human and animal models are associated with altered NMDAR trafficking and NMDAR hypofunction in the limbic system. This has been partly supported by evidence of decreased expression of NMDAR subunits and associated proteins in the brains of schizophrenic patients relative to controls [156, 157]. Thus, the dysregulation of NMDAR trafficking might contributes to the etiology of schizophrenia [140, 158].

Several genes associated with schizophrenia regulate NMDAR trafficking or activation. These genes include neuregulin, PP2B calcineurin *γ*-subunit, N-acetyl aspartyl glutamate- (NAAG-) related genes, glutamate carboxypeptidase II (GCPII), and metabotropic glutamate receptor 3 (mGluR3). Neuregulin-1 (NRG1), a growth factor genetically linked to schizophrenia in humans, promotes rapid internalization of NMDARs from the cell surface by a clathrin-dependent mechanism in prefrontal pyramidal neurons [159, 160]. NRG1 acts at its receptor, ErbB4, to modulate NMDAR signaling [161]. In human prefrontal cortex, NRG1 stimulation causes a stronger suppression of NMDAR activation in patients with schizophrenia, due to an enhanced interaction between ErbB4 and PSD-95 [161]. Moreover, overactivation of the ErbB4 receptor by neuregulin suppresses tyrosine phosphorylation of GluN2A in the prefrontal cortex of patients with schizophrenia and could suppress NMDAR activity, eliciting schizophrenia-like symptoms [161].

Another candidate schizophrenia gene, calcineurin PP2B *γ*-subunit (PPP3CC), promotes NMDAR internalization via STEP [161–163]. This gene, located at 8p21.3, was identified in families affected with schizophrenia [161–163]. In the caudate nucleus of postmortem schizophrenia patients, tissue immunoreactivity for calcineurin is increased with respect to control patients [164]. Studies examining biomarkers for schizophrenia, specifically in whole blood, have found increased RNA expression of calcineurin in patients with schizophrenia. Calcineurin has therefore become an effective predictor for progression of this disease [165].

Several studies have demonstrated that the expression of NAAG-related genes, GCPII and mGluR3, are reduced in the dorsolateral prefrontal cortex and hippocampus in schizophrenia. NAAG is an endogenous mGluR agonist and NMDAR antagonist. NAAG is a peptide neurotransmitter found in high concentrations in the mammalian brain. It is concentrated in synaptic vesicles, released upon depolarization in a calcium-dependent manner and metabolized by GCPII, a membrane-bound peptidase. Thus, a reduction in GCPII expression would result in an increase in NAAG in hippocampal cells of patients or models schizophrenia. NAAG has been shown to preferentially affect NMDARs. In rodent CA1 pyramidal neurons, increasing the concentration of NAAG resulted in a significant reduction in the NMDAR component of evoked excitatory postsynaptic currents (EPSCs) [166, 167].

Pharmacologically, the relationship between NMDARs and schizophrenia is demonstrated in recent studies where phencyclidine (PCP), a noncompetitive antagonist of NMDARs, has been used as a pharmacological model of schizophrenia in rats/rodents. PCP binds to a site within the pore of the channel that is only accessible when the channel is open; therefore, the antagonism is “use-dependent.” PCP was first developed as a surgical anesthetic. Despite its efficacy as an anesthetic, widespread clinical use was not possible because, after surgery, patients experienced hallucinations, disordered speech, delirium, agitation, and disoriented behavior similar to symptoms reported in patients with schizophrenia. Indeed, evidence suggests that PCP can be used in rodents to produce a pattern of metabolic, neurochemical, and behavioral changes similar to those seen in patients with schizophrenia [168]. This has given considerable insight into the processes that underlie the etiology of the disease, highlighting the potential importance of NMDAR hypofunction.

A study investigating the relationship between TBI and schizophrenia showed that patients with TBI exhibited symptoms of psychotic disorders easily confused with schizophrenia following injury [169]. In general, patients with psychotic disorders triggered by a TBI event show fewer negative symptoms and also show a positive finding in MRI/CT studies (lesions are more localized) [169]. In these cases, family history of schizophrenia is a risk factor, particularly in males [169]. However, similar to other conditions described, the mechanism underlying the relationship between the chronic pathology and the acute event is unknown.

### 5.3. Parkinson's Disease

PD is the most common movement disorder characterized by resting tremor, rigidity, bradykinesia, and postural instability. These clinical features are thought to result from reduced dopaminergic input to the striatum, which is caused by the loss of dopaminergic neurons in the substantia nigra [170]. The occurrence of PD is largely sporadic, but familial PD has been linked to mutations in at least 5 distinct genes (*α*-synuclein, parkin, DJ-1, PINK1, and LRRK2). Parkin, DJ-1, and PINK1 gene products are mitochondrial proteins required to protect neurons from reactive oxygen species (ROS) and have been shown to be necessary to protect against dopamine-mediated oxidative stress [171]. Because dopamine induces oxidative stress in neurons, the loss of neuroprotective proteins can render dopaminergic neurons particularly vulnerable to oxidative stress [172].

PD pathophysiology is linked to a widespread process of degeneration of dopamine-secreting neurons in the substantia nigra pars compacta, with the consequent loss of the neurons projecting to the striatum [173, 174]. In the striatum as well as in other brain areas, LTP requires activation of NMDARs [175]. Interestingly, it has become increasingly evident that, in striatal spiny neurons, the NMDAR complex is also profoundly altered in experimental preclinical PD animal models (rats and mice) [176]. Early studies evaluated NMDAR abundance, composition, and phosphorylation in models of PD. In the dopamine-denervated striatum, a decreased level of GluN1 and GluN2B subunits has been found in striatal membranes, while the abundance of GluN2A was unchanged [176, 177]. Moreover, binding of GluN2B to SAP102 and SAP97 (MAGUKs proteins, which regulate the delivery of the NMDAR subunit to the membrane) is significantly reduced in dopamine-denervated rats leading to the reduction of GluN2B protein levels in the postsynaptic density [176, 177].

It is well established that the phosphorylation state of NMDARs regulates their functional characteristics, subcellular distribution, and anchoring to the plasma membrane in physiological and pathological conditions. CaMKII (Ca^2+^/calmodulin-dependent protein kinase II), crucial for synaptic plasticity, and tyrosine-dependent phosphorylation of NMDARs increases after nigrostriatal denervation, leading to receptor sensitization [178, 179]. Moreover, NMDAR subunits GluN2A and GluN2B interact with membrane-associated guanylate kinases (MAGUK); this interaction governs their trafficking and clustering at synaptic sites [180]. In a model of L-DOPA-induced dyskinesia, PSD-95, SAP97, and SAP102 are reduced in the postsynaptic compartment in rats with Parkinson-like pathology compared with sham-operated rats [181]. These animals have significantly higher levels of GluN2A subunits in the postsynaptic site, and the levels of GluN2B subunits are significantly reduced. This evidence suggests that the trafficking of GluN2 subunits may be altered in early stages of PD. Using a peptide to disrupt the interaction between GluN2B and MAGUK proteins, the localization of GluN2B-containing NMDAR is altered and allows modulating the dyskinetic motor behavior in an animal model of dyskinesia [181]. Thus, developing therapies that regulate the trafficking of GluN2 may alleviate PD symptoms. It is possible to have a combination of genetic and external factors contributing to the onset of PD. It has been found that patients with high levels of synuclein prior to experiencing head trauma initiate and/or accelerate neurodegeneration observed in PD [182] but the molecular mechanisms behind these events are unknown.

There is evidence that TBI can contribute to disease development and/or progression of PD. A study with 52,393 TBI patients and 113,406 control patients showed that TBI is associated with a 44% risk of developing PD over 5 to 7 years after injury [183]. Another study, analyzing the medical record of 196 subjects who developed PD (between 1976 and 1995), found that the PD group had significantly more events of head trauma than controls [184]. In this study the authors proposed three different alternatives to the genesis of PD after a single brain trauma: first, the neuronal loss in the substantia nigra could produce a predisposition to later development of PD; second, brain trauma could disrupt the blood-brain barrier allowing the introduction of immunological mediators; third, head trauma could trigger the expression and later deposition of misfolding proteins in Lewy bodies, similar to what occurs with A*β* in AD [184]. More recently, a meta-analysis reviewing literature indicates that a history of head trauma, resulting in contusion, is associated with higher risk of developing PD [185]. Research in twins showed that mild-moderate closed-head injury may increase the risk for PD even decades after the brain injury episode [186]. It is possible to apply the model in Figure 2(b) for the onset of PD. Here, TBI accelerates the progression of neurodegeneration causing the appearance of symptoms at an earlier age.

### 5.4. Huntington's Disease

HD is a progressive neurological disorder caused by an autosomal dominant mutation. Symptoms of HD include abnormal writhing, dance-like movements, cognitive disturbances, and disorders of mood, many of which precede onset of the motor abnormalities [187]. HD is characterized by striatal dysfunction and neurodegeneration that is caused by a polyglutamine expansion in the protein huntingtin (Htt) [188]. The CAG repeat expansion in the gene encoding the protein Htt produces 35 polyglutamine repeats or more leading to HD, with longer repeats being associated with earlier disease onset. Both Htt and mutant Htt (mHtt) are ubiquitously expressed in the brain; the highest levels are found in the cerebellum, a region spared in HD, whereas levels in the striatum are comparatively low [189].

Cognitive disturbances present in HD patients long before the onset of overt motor manifestations [190]. Furthermore, neuronal and synaptic dysfunction precede cell death by many years in humans and occur long before [191, 192], or in the absence of, cell death in HD animal models [193]. NMDAR currents, surface expression, and excitotoxicity are enhanced between 9 and 11 weeks in HD transgenic mice [194], and the function and trafficking of NMDARs that contain the GluN2B subunit are altered [195]. Synaptic NMDARs activate prosurvival pathways, while extrasynaptic NMDARs trigger cell death [34]. A shift in the balance of synaptic to extrasynaptic NMDAR signaling contributes to HD pathology, as chronic extrasynaptic NMDAR blockade attenuates mHtt-induced striatal atrophy and motor learning deficits in YAC128 mice, transgenic mice that express the human huntingtin protein containing a 128 CAG repeat expansion [195, 196]. Furthermore, along with elevated extrasynaptic NMDARs activity, intracellular Ca^2+^ signaling pathways that couple to survival or death are also deregulated early in HD. Activity of the Ca^2+^-dependent protease calpain is elevated in striatal tissue of postmortem HD human brains and presymptomatic 1-2-month-old YAC128 mice [197, 198]. In brief, calpain potentiates HD-associated striatal degeneration by cleaving mHtt into toxic fragments and triggering proapoptotic cascades in parallel with caspases [199, 200]. In addition, activity of the prosurvival transcription factor CREB is reduced in striatal tissue of 1- and 4-month-old YAC128 mice [195]. While synaptic NMDAR signaling promotes CREB activity, extrasynaptic NMDARs trigger dephosphorylation and inactivation of CREB [34]. Additionally, CREB signaling is restored by chronic suppression of extrasynaptic NMDAR activity in YAC128 mice [195], suggesting a link between extrasynaptic NMDARs and CREB inactivation. Both increased extrasynaptic NMDAR activity and deregulated intracellular signaling could contribute to mHtt-induced striatal degeneration.

Pharmacologically, Dimebon has been proposed as a tool in the treatment of neurological diseases including HD [201]. One of the possible mechanisms of action of Dimebon is to inhibit NMDAR activity [201].

While there are no reports of an association between HD and TBI, the glutamatergic hypothesis fits with the mechanism underlying HD, and it is possible that TBI could be an aggravating event in those with a risk for developing HD.

### 5.5. Amyotrophic Lateral Sclerosis

ALS is a fatal neurodegenerative disease characterized by muscle atrophy, weakness, and fasciculation indicative of a disease of the upper and lower motor neurons (MNs). Lateral sclerosis refers to the hardness of the spinal cord lateral columns in autopsy specimens, due to the massive gliosis caused by the degeneration of corticospinal tracts [202]. ALS occurs in both familial (fALS, 10%) and sporadic (sALS, 90%) forms that are clinically indistinguishable. A growing number of ALS-causing genes have been identified and are under investigation [203]. The ubiquitously expressed enzyme Cu^2+^/Zn^2+^ superoxide dismutase (SOD) was the first of such genes to be associated with ALS [204]. SOD1 mutations are common in both fALS and sALS and have been studied in the most depth. Over 150 SOD1 mutations have been linked to fALS and are typically present in about 20% of such cases and are present in the 7% of sALS cases [205]. Other genes identified in fALS are alsin (ALS2) [206], senataxin (ALS4) [207], and vesicle associated membrane protein associated protein B (VAPB, ALS8), to name a few [208]. The animal models of ALS are transgenic mice carrying mutant SOD1 (mSOD1); these animals have been a valuable tool to study the pathological mechanism underlying the disease progression and the degeneration of MNs [209].

The first indication that glutamate neurotransmission was linked to the pathogenesis of ALS was the discovery of elevated glutamate levels in the plasma and cerebrospinal fluid (CSF) of ALS patients compared to healthy controls [210]. Following these first observations, a decrease in the maximal velocity of transport for the high affinity glutamate uptake in spinal cord synaptosome preparations from ALS patients was described [211]. In physiological conditions, motor neurons, surrounded by resting astrocytes, receive synaptic glutamatergic inputs by the descending fibers. Glutamate released by the presynaptic neuron stimulates its receptors on the postsynaptic neuron generating excitatory postsynaptic potentials, contributing to neuronal plasticity. The action of the neurotransmitters is ultimately terminated by the intervention of the glial glutamate transporters that then take up glutamate into astrocytes [212, 213]. In ALS, presynaptic hyperexcitability generates excessive glutamate release from the presynaptic neuron [214]. In addition, the simultaneous occurrence of a reduced expression of the glial glutamate transporter GLAST/GLT1 determines a pathological increase in the extracellular concentrations of glutamate in the synaptic cleft [215]. This produces an overstimulation of the glutamate receptors on the postsynaptic neurons with a consequent cellular excitotoxicity (see mechanism below) on top of concurrent factors such as mitochondrial failure and endoplasmic reticulum stress [216, 217]. The occurrence of all these events leads to cell death.

Studies of spinal neuronal excitability in ALS thus far have been restricted to cell culture and neonates and have quite exclusively looked at AMPAR-mediated currents and neuronal hyperexcitability mainly mediated by Na^+^ currents. However, the contribution of NMDARs to ALS pathology was demonstrated in mSOD1 mice in a study investigating whether NMDARs play a role in the increased bursting activity generated by spinal interneurons [218]. Their results indicate that NMDARs on spinal interneurons are a potential source of overexcitation of MNs as the disease progresses. The overactivation of NMDARs results in mitochondrial membrane depolarization and opening of the mitochondrial permeability transition pore (mPTP), ROS production, and caspase activation [219]. Mitochondrial Ca^2+^ accumulation and the subsequent release is a critical step in acute glutamate excitotoxicity [216, 220], leading to failure to maintain intraneuronal Ca^2+^ concentrations. Excessive Ca^2+^ influx through NMDARs impacts mitochondria which then trigger apoptosis cascades that result in ALS-related MN death [221, 222]. Mitochondria-mediated apoptosis has been linked to MN degeneration and the involvement of the mPTP has been shown to be an active player in the mechanisms of MN death in ALS [223, 224]. Accordingly, mPTP-driven glutamatergic excitotoxicity has been observed in both spinal glycinergic interneurons and MNs and has been associated with ALS neurodegeneration [225]. The Ca^2+^-mediated excitotoxicity in neurofilament aggregate-bearing MNs* in vitro* is primarily an NMDAR-dependent process and requires caspase-3 activation [226]. The sequential activation of caspase-1 and caspase-3 has been observed in MNs and astrocytes bearing the mSOD1 forms [227]. These observations support the model for a glutamatergic/excitotoxic mechanism in ALS.

A meta-analysis of the relationship between ALS and head trauma found that repetitive head injury was related to a higher risk of ALS in the American population [228] though a recent study did not find an association between head injury and ALS. In this study, a linear regression was performed to determine if head injury was a predictor for the mean monthly decline of ALS using the Functional Rating Scale-Revised (ALSFRS-R) [229]. In this study, 24 ALS patients with TBI and 76 ALS control patients were compared. Described brain lesions for tau pathology and AD also show not differences between groups [229], generating controversial results. Another very recent report found after a meta-analysis that mild TBI is associated with development of neurological diseases including ALS [230]. More studies will be needed to examine the relationship between TBI and neurological diseases.

### 5.6. Major Depressive Disorder

Major depressive disorder (MDD) is a psychiatric disorder that affects millions of people worldwide. Individuals battling this disorder commonly experience high rates of relapse, persistent residual symptoms, functional impairment, and diminished well-being. Depressed patients have high susceptibility for suicide, in part due to complications arising from stress [231]. Medications have important utility in stabilizing moods and daily functions of many individuals. However, only one-third of patients show considerable improvement with a standard antidepressant after two months and these medications are associated with a number of side effects [232, 233].

Current medications for depression increase the level of biogenic amines such as norepinephrine (NE), dopamine (DA), and serotonin (5-HT) by a variety of mechanisms including inhibiting the degradation or blocking reuptake of the neurotransmitters [231, 234, 235]. NMDARs have received special attention because of their critical role in psychiatric disorders [236]. Antidepressant-like effects have been demonstrated by several types of NMDAR antagonists in different animal models [237]. These antagonists include competitive and noncompetitive antagonists and partial agonists at strychnine insensitive glycine receptors, and antagonists acting at polyamine binding sites. MK-801 (a use-dependent channel blocker or noncompetitive antagonist) and CGP 37849 (a competitive antagonist) have shown antidepressant properties in preclinical studies, either alone or combined with traditional antidepressants [236, 238, 239]. Furthermore, ketamine is noncompetitive NMDARs antagonist and a derivative of PCP which was found to produce rapid, robust, and persistent antidepressant effects clinically [240, 241]. Therefore, it appears that NMDAR antagonists may be key to developing a new generation of improved treatments for major depression.

Several studies in children who have suffered from head trauma show several disorders even in adulthood [242]. The spectrum of disorders includes hyperactivity, conduct problems, and social and emotional issues such as anxiety and depression [243, 244]. Several studies have identified TBI as a risk factor for developing depressive disorders [245, 246]. Some pharmacological approaches have been used [247–249] but the cellular mechanism underlying the brain alterations has not been addressed.

## 6. Conclusions and Remarks

In this review, we have discussed the current literature on the consequences of TBI in the context of several neurological conditions. Though little is known about the cellular mechanisms involved, the contribution of NMDARs to disease progression seems to link these diverse diseases with common consequences given the role of NMDARs in excitotoxicity, neuronal, and synaptic damage. The majority of information available focuses on AD and PD, but research highlighting the role of glutamatergic transmission has identified associations with the glutamate hypothesis to several other diseases.

The paradoxical contribution of NMDARs to physiopathological events, the balance between the beneficial and deleterious effects, appears to be a very attractive focus in the search for molecular target for several neurological diseases.

In this review, we focused our attention on representative chronic and acute conditions where NMDARs and glutamatergic transmission have a role controlling the final destination of injured neurons highlighting the common mechanism of toxicity of acute conditions and chronic pathologies (Figure 2(a)). We have reviewed the biomedical background to demonstrate that traumatic brain injury is able to generate damage that contributes to the early onset of subjacent chronic diseases (Figure 2(b)). We propose that NMDAR distribution plays a key role in the aggravation of these chronic diseases. Alteration in the distribution of NMDARs in AD, PD, HD, and ALS is the precipitates of the extensive damage after TBI. Extrasynaptic NMDARs are available in high amounts ready to be activated by glutamate spillover leading to calcium overload associated with excitotoxicity, neuronal damage, and death. The signaling implicated in intermediate steps is also altered, specifically signaling mediated by calcium and the crosstalk with multiple pathways, providing a battery of putative therapeutic targets to modulate the neurotoxicity mediated by NMDARs.

## Figures and Tables

**Table 1:** Proteins involve in location-dependent NMDAR signaling activation.

Protein	Location of stimulated NMDA receptor	NMDA receptor activation induces ↑ or ↓	Closest partners upstream	Reference
CREB	Syn	↑	CBP phosphorylation by CaMKIV	[42, 43]
	Extrasyn	↓	CREB dephosphorylation by Jacob	[39]

ERK 1/2 (MEK phosphorylation)	Syn	↑	Ras-GTP	[32, 44]
	Extra	↓	Ras-GDP by SynGAP	

Ras	Syn	↑	Increase in Ca^2+^ Ras-GTP	[32, 44]
	Extrasynaptic	↓	Less Ca^2+^ Ras-GDP	

FOXO1/FOXO3 (transcription factor, apoptosis inducer)	Syn	↓	FOXO1/3 phosphorylation by akt inducing nuclear export	[35]
	Extrasynaptic	↑	FOXO3a nuclear translocation	[45]

Calpain	Extrasynaptic	↑	Calpain Ca^2+^ activated	[46]

STEP	Extrasynaptic	↑	STEP cleaved by calpain	[36]

NCX3	Extrasynaptic	↑	NCX3 cleaved by calpain	[21]

Akt/PI3K	Syn	↑	Akt/PI3K activated through IRS-1	[35, 47, 48]

SynGap	Syn	↑	Activated by CamKII	[49]

CaMKII	Syn	↑	Increase in Ca^2+^	[50]
